# Preoperative Prediction of Extramural Venous Invasion in Rectal Cancer: Comparison of the Diagnostic Efficacy of Radiomics Models and Quantitative Dynamic Contrast-Enhanced Magnetic Resonance Imaging

**DOI:** 10.3389/fonc.2020.00459

**Published:** 2020-04-09

**Authors:** Xiangling Yu, Wenlong Song, Dajing Guo, Huan Liu, Haiping Zhang, Xiaojing He, Junjie Song, Jun Zhou, Xinjie Liu

**Affiliations:** ^1^Department of Radiology, The Second Affiliated Hospital of Chongqing Medical University, Chongqing, China; ^2^GE Healthcare, Shanghai, China

**Keywords:** rectal cancer, extramural venous invasion, radiomics, dynamic contrast-enhanced magnetic resonance imaging, quantitative parameters, prediction

## Abstract

**Background:** To compare the diagnostic performance of radiomics models with that of dynamic contrast-enhanced magnetic resonance imaging (DCE-MRI) perfusion parameters for the preoperative prediction of extramural venous invasion (EMVI) in rectal cancer patients and to develop a preoperative nomogram for predicting the EMVI status.

**Methods:** In total, 106 rectal cancer patients were enrolled in our study. All patients under went preoperative rectal high-resolution MRI and DCE-MRI. We built five models based on the perfusion parameters of DCE-MRI (quantitative model), the radiomics of T_2_-weighted (T_2_W) CUBE imaging (R_1_ model), DCE-MRI (R_2_ model), clinical features (clinical model), and clinical-radiomics features. The predictive efficacy of the radiomics signature was assessed and internally verified. The area under the receiver operating curve (AUC) was used to compare the diagnostic performance of different radiomics models and DCE-MRI quantitative parameters. The radiomics score and clinical-pathologic risk factors were incorporated into an easy-to-use nomogram.

**Results:** The quantitative parameters *K*^*trans*^ and Ve were significantly higher in the EMVI-positive group than in the EMVI-negative group (both *P* =0.02). *K*^*trans*^ combined with Ve showed a fair degree of accuracy (AUC 0.680 in the training cohort and AUC 0.715 in the validation cohort) compared with *K*^*trans*^ or Ve alone. The AUCs of the R_1_ and R_2_ models were 0.826, 0.715 and 0.872, 0.812 in the training and validation cohorts, respectively. In addition, the R_2_-C model yielded an AUC of 0.904 in the training cohort and 0.812 in the validation cohort. The nomogram was presented based on the clinical-radiomics model. The calibration curves showed good agreement.

**Conclusion:** The radiomics nomogram that incorporates the radiomics score, histopathological grade and T stage demonstrated better diagnostic accuracy than the DCE-MRI quantitative parameters and may have significant clinical implications for the preoperative individualized prediction of EMVI in rectal cancer patients.

## Introduction

Rectal cancer is the third most common malignant cause of morbidity and mortality globally (1). Local recurrence and metastasis are the major causes of death in patients with rectal cancer. Extramural venous invasion (EMVI), defined as “the presence of tumor cells within blood vessels beyond the muscularis propria” (2), which is present in 31% of patients with rectal cancer (3), is one of the main factors that affect the risk of recurrence (4) and an independent indicator for a poor prognosis (5–7). Therefore, the identification of EMVI is critical for accurate preoperative risk stratification and influences decision-making (8, 9).

Traditionally, postoperative pathology diagnosis is considered the gold standard for EMVI. Recently, high spatial and contrast resolution with magnetic resonance imaging (MRI) has been shown to provide moderate to high specificity and accuracy for the preoperative assessment of EMVI. However, there are still many problems associated with human assessment based on MRI, such as heterogeneity with image quality, methods and diagnostic accuracy (10) and very small (≤3 mm) tumor deposits within vessels that are beyond the resolution limits of MRI (11). Furthermore, the inflammation, edema, and fibrosis caused by neoadjuvant chemoradiotherapy (CRT) may also affect the human assessment of EMVI on MRI (10, 12, 13). Additionally, some studies have reported a relatively low and wide range of sensitivity (28.2–62%) of EMVI evaluation on conventional MRI (6, 14, 15). Therefore, an accurate, objective and non-invasive preoperative method for EMVI evaluation is still needed.

Dynamic contrast-enhanced magnetic resonance imaging (DCE-MRI) is a non-invasive functional imaging technique used to reflect the attributes of tumor microcirculation that integrates morphology and changes in hemodynamics (16). Since DCE-MRI has the ability to quantify parameters related to tumor micro angiogenesis, perfusion, and permeability (17, 18), it has been widely used for rectal cancer detection, staging, prediction, assessment of responses to neoadjuvant treatment, tumor angiogenesis, biologic aggressiveness, and molecular markers (19–22). To our knowledge, there are few studies on DCE-MRI for the evaluation of EMVI in rectal cancer. Recently, Chen et al. reported an association between MRI-detected EMVI and DCE-MRI parameters in rectal adenocarcinoma and demonstrated that mrEMVI-positive patients had significantly lower Kep and higher Ve values than mrEMVI-negative patients (23). However, the diagnostic efficacy of DCE-MRI quantitative parameters is still unclear.

Radiomics, an emerging field in radiology that quantifies imaging data and involves the high-throughput extraction of a large number of quantitative features from medical images, has recently attracted growing attention, providing non-visual information related to tumor heterogeneity that can be used in personalized treatment (24, 25). Regarding rectal cancer, the potential application of radiomics mainly involves the prediction of the response after CRT, survival, and other pathological features, such as T stage, lymph node metastasis, and perineural invasion (26–30). However, to our knowledge there is no study that has determined whether a radiomics signature could enable the prediction of EMVI.

Therefore, our study aimed to construct and validate different radiomics models for the preoperative prediction of EMVI in patients with rectal cancer and to compare the diagnostic performance of these models with perfusion parameters based on DCE-MRI, developing a radiomics nomogram for the accurate preoperative risk stratification and selection of adjuvant therapy or individualized treatment.

## Materials and Methods

### Patients

This retrospective study was approved by the Medical Ethics Committee of the Second Affiliated Hospital of Chongqing Medical University, and all patients provided written consent for the DCE-MRI examination.

A total of 230 consecutive patients with pathologically confirmed rectal cancer by biopsy or surgery from November 2016 to May 2019 were included in this study. All patients underwent preoperative rectal high-resolution MRI and DCE-MRI. The inclusion criteria were as follows: (1) histologically confirmed rectal adenocarcinoma and (2) no history of pelvic surgery. Patients were excluded from the analysis for the following reasons: (1) patients did not undergo surgery (*n* = 37), (2) insufficient MRI quality for measurements (*n* = 16), (3) incomplete scanning sequence for MRI (*n* = 19), (4) lesions were too small to be accurately measured (*n* = 20), (5) proven special histopathological type, including mucinous adenocarcinoma, signet ring cell carcinoma, and sarcomatous carcinoma (*n* = 9),and (6) lesions with obvious irregular vessel contour or nodular expansion of vessel with definite tumor signal that scored 4, based on the five-point grading system suggested by Smith et al. (31) (*n* = 23) (Supplementary Figure A1).

Ultimately, 106 patients were enrolled in the study. Baseline clinical-pathological prognostic factors, including age, sex, tumor location, tumor size, histological grade, histological tumor stage, lymph node stage, carcinoembryonic antigen (CEA) level, and Ki-67, were derived from the patients' electronic medical records.

### Histopathologic Evaluation

Histopathological information, including T stage, N stage, histological grade, and the presence of EMVI, was obtained from pathology reports and confirmed by a pathologist with 5 years of experience in pathology. Lymph node stage was categorized as negative (no metastasis in regional nodes) or positive (including N1, metastasis in 1–3 nodes; and N2, metastasis in four or more nodes). Histological grade was categorized into three stages (well, moderately, and poorly differentiated). The pathologic definition of EMVI was the presence of a rounded mass of tumor tissue within an endothelium-lined space beyond the muscularis propriain H&E-stained slides. A pathologic result of EMVI (present, absent) was obtained for each patient.

### MRI Acquisition

MRI scanning was performed using a 1.5-T MR scanner (GE HDXT2012, USA) with an eight-element pelvic phased-array body coil. To reduce intestinal peristalsis or rectal spasm, 20 mg of hydrochloride hyoscine butylbromide (raceanisodamine, Hangzhou Minsheng Pharmaceutical Co., Ltd., China) was injected intramuscularly 10–15 min before MRI unless contraindicated. Patients were instructed to fast for 12 h before the examination and to empty the contents of the intestine.

Non-enhanced MRI, including routine oblique axial T_1_-weighted (T_1_W) imaging, fat-saturated respiratory-triggered fast recovery fast spin-echo (FRFSE) T_2_W imaging, oblique axial T_2_W CUBE imaging, and sagittal high-resolution turbo spin-echo (TSE) T_2_W imagingwere obtained. DCE-MRI was performed following non-enhanced sequences. Before injection of the contrast agent, the same oblique axial 3D liver acceleration volume acquisition (LAVA) gradient-echo T_1_W sequence with five different flip angles (2, 5, 8, 12, and 15°) was used to obtain T_1_ mapping images. Then, the contrast agent, gadolinium-diethyl enetriaminepentacetate (Gd-DTPA, Omniscan GE Healthcare Life Science; 0.2 mmoL/kg) was injected through the cubital vein at 2.5 mL/s using high-pressure syringes (Spectris MR Injector System; Medrad) followed by 20 mL of normal saline flush at the same rate in the third period of the dynamic scan. The DCE-MRI scanned 52 slices during each phase, and a total of 32 periods of uninterrupted scanning were performed. The scanning time was 5 min and 10 s. The parameters of all MRI protocols are shown in Table 1.

**Table 1:** MRI sequences and parameters.

**Parameter**	**T_**1**_W**	**T_**2**_W FRFSE**	**Sagittal T_**2**_W**	**Oblique axis T_**2**_W CUBE**	**DCE**
Repetition time (ms)	780	4,140	4,240	2,000	3.9
Echo time (ms)	8.6	72.5	102	103	1.3
Flip angle (degrees)	n/a	n/a	n/a	n/a	15
Field of view (mm)	260 × 260	260 × 260	260 × 260	260 × 260	400 × 320
Slice thickness (mm)	6	6	5	2	5
Slice gap (mm)	1	1	1	0	0
Fat saturation	No	Yes	No	No	Yes
Base matrix	320 × 192	288 × 192	320 × 192	224 × 224	224 × 160

*T1W, T1-weighted; T2W FRFSE, T2-weighted fat-saturated respiratory-triggered fast spin-echo; T_2_W CUBE, T2-weighted CUBE; DCE, dynamic contrast-enhanced*.

### Image Post-processing and Analysis

#### DCE-MRI

Image assessment was performed for each patient by a consensus of two abdominal radiologists with at least 5 years of experience in pelvic MRI images, blinded to the clinical and pathologic outcomes.

For the measurement of DCE-MRI parameters, a pharmacokinetic analysis was carried out using Omni kinetics software (OK, GE Healthcare, China) with the two-compartment extended Tofts model. T_1_ maps were generated using five different flip angles (2, 5, 8, 12, and 15°). The arterial input function (AIF) was derived from the left external iliac artery. Then, the time-concentration curve was calculated.

**Figure 1:**
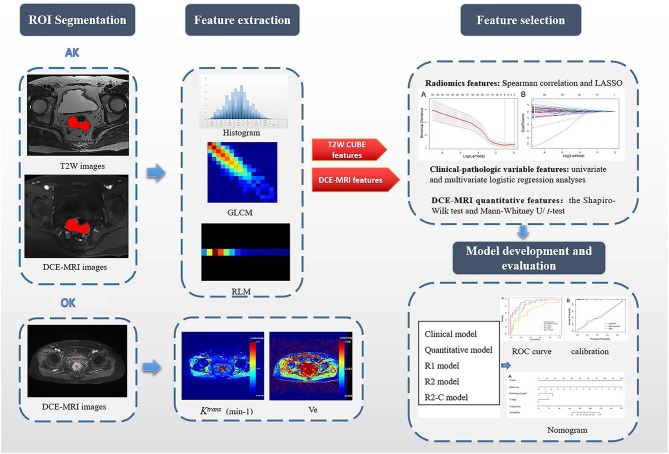
Flowchart of this study. First, after the VOIs/ROIs of the tumor were manually segmented, 396 features were first extracted each from T_2_W imaging and DCE imaging. For the measurement of DCE-MRI parameters, a pharmacokinetic analysis was carried out using OK software with the two-compartment extended Tofts model. Second, LASSO analysis was used to reduce the redundancy or selection bias of the features. Then, the Rad-score was calculated for each patient using a linear combination of selected features that were weighted by their respective coefficients. Thereafter, the most significant features for the prediction of EMVI were investigated to construct the radiomics model on the basis of logistic regression; clinical-pathologic risk factors were compared via univariate and multivariate analyses; the quantitative features in DCE-MRI were selected by the Mann–Whitney U/*t*-test; Finally, different models were constructed and compared. A radiomics nomogram based on the clinical-radiomics model was constructed. Calibration curves and the Hosmer-Lemeshow test were used to graphically investigate the performance characteristics of the radiomics nomogram that were tested in the validation cohort. GLCM, gray-level co-occurrence matrix; RLM, run-length matrix; *K*^*trans*^, volume transfer constant between the blood plasma and the extracellular extravascular space; Ve, extracellular extravascular space volume fraction; R_1_ model, radiomics models based on T_2_W CUBE; R_2_ model, radiomics models based on DCE; R_2_-C model, the combined model based on DCE and clinical-pathological factors.

Two radiologists independently assessed the images and manually drew tumor regions of interest (ROIs) along the edges of the tumor slice by slice for the entire tumor while avoiding visible blood vessels, peripheral fat, areas of necrosis, or hemorrhage, and the intestinal lumen as far as possible (Figure 1). Then, all ROIs were merged for the whole tumor volume ROI.

The estimated kinetic model quantitative parameters were as follows: volume transfer constant between the blood plasma and the extracellular extravascular space (*K*^*trans*^), extracellular extravascular space (EES) volume fraction (Ve), plasma volume fraction (Vp), and rate constant of contrast agent escape from the EES into the plasma compartment (Kep). The semiquantitative parameters, including the initial area under the enhancement curve (iAUC), time to peak (TTP), max slope and max concentration, were calculated from concentration-time curves derived from the entire ROI for each patient.

#### MRI Radiomics

##### Image segmentation and radiomics feature extraction

Volumes of interest (VOIs) were manually segmented on T_2_W CUBE imaging and during the fifth phase of DCE imaging (60 s after injection of the contrast agent) via a free open source software package (ITK-SNAP, version 3.6.0, covering the whole tumor were delineated along the border of the tumor and excluding the intestinal lumen. All tumor segmentations were confirmed by a senior radiologist with 10 years of experience in pelvic imaging. Next, the segmented tumor VOI files were imported into in-house software (Artificial Intelligence Kit, AK, version 3.2.2, GE Healthcare) for texture analysis. Image registration for MR image was also performed on the AK software, and every MR image was resampled to a uniform pixel dimension of 1.0 × 1.0 × 1.0 mm^3^ before feature extraction. The delineation of the workflow is shown in Figure 1.

Radiomics features were calculated automatically with AK software. In total, 792 features were extracted for each patient: 396 features each from T_2_W and DCE imaging. These radiomics features were divided into five groups: (1) intensity histogram features; (2) morphological features; (3) gray-level co-occurrence matrix (GLCM) features; (4) gray-level run-length matrix (RLM) features; and (5) gray-level size zone matrix (GLSZM) features.

##### Feature selection and radiomics signature building

**Figure 2:**
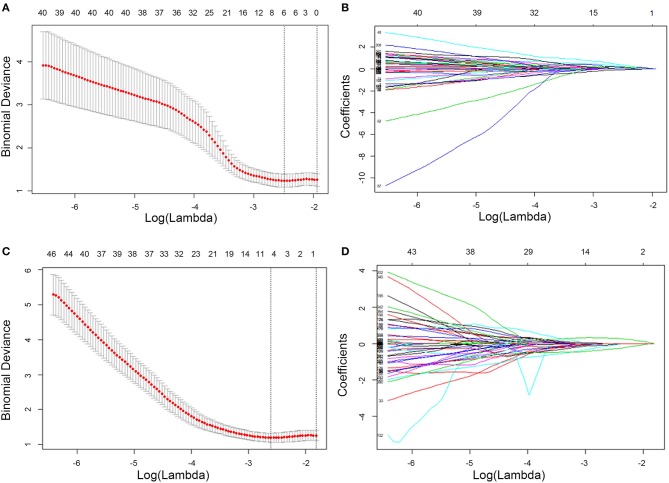
Feature selection using LASSO logistic regression of T2W CUBE **(A,B)** and DCE **(C,D)** imaging. LASSO coefficient analysis of the 792 radiomics features. A 10-fold cross-validation was applied with the regularization parameter (λ) of the LASSO regression model and selected when the deviance was minimal **(A,C)**. Coefficients are plotted against the log (λ) sequence. Ultimately, four nonzero coefficients were selected **(B,D)**. LASSO, least absolute shrinkage and selection operation.

Spearman correlation (r threshold of 0.9) and least absolute shrinkage and selection operator (LASSO) analyses were used to reduce the redundancy or selection bias of the features in the training cohort. When two features are highly correlated, the feature of greater contribution was retained. A 10-fold cross-validation was applied with the regularization parameter (λ) of the LASSO method and was selected when the deviance was minimal (Figure 2). Backward stepwise selection was applied, and the stopping rule was the likelihood ratio test with Akaike's information criterion. Finally, the most significant features for the prediction of EMVI were investigated to construct the radiomics model on the basis of logistic regression. The radiomics score (Rad-score) was calculated for each patient using a linear combination of selected features that were weighted by their respective coefficients.

##### Development and validation of the prediction model

Clinical-pathologic risk factors were compared via a univariate analysis; variables with *P* < 0.05 were included in the clinical model. The quantitative parameters in DCE-MRI were selected by the Mann–Whitney *U*-test (variables with non-normal distribution) or the *t*-test (variables with normal distribution). The most significant parameters were used to build the quantitative model. In addition, three different radiomics models, individual T_2_W CUBE (R_1_ model), DCE (R_2_ model) and clinical-radiomics were built using a multivariable logistic regression analysis.

##### Construction and validation of the radiomics nomogram

The comparisons of predictive performance were evaluated using the DeLong test. To offer an individual and visual tool for predicting the probability of EMVI, we constructed a radiomics nomogram based on the clinical-radiomics model. Calibration curves and the Hosmer–Lemeshow test were used to graphically investigate the calibration characteristics of the radiomics nomogram, which were tested in the validation cohort.

### Statistical Analysis

The Shapiro–Wilk test was used to determine whether the variables were normally distributed. The *t*-test or the Mann–Whitney *U*-test was performed to compare continuous variables, while a chi-squared test or Fisher's exact test was used to classify variables between groups. Univariate and multivariate logistic regression analyses were used to identify independent predictors of EMVI. The discrimination performance of the radiomics models was quantified by the area under the receiver operating curve (AUC) value in the training cohort and internally validated in the independent validation cohort. The diagnostic efficacy of the clinical, quantitative, and radiomics models was compared based on the AUC, sensitivity, and specificity.

Interobserver agreement for DCE-MRI quantitative perfusion parameter measurements between the two radiologists was analyzed by calculating the intraclass correlation coefficient (ICC) with the 95% confidence interval (CI).

Statistical analyses were performed with R software (version 3.6.0, http://www.Rproject.org). Two-sided *P* < 0.05 were considered statistically significant.

## Results

### Clinical-Pathologic Characteristics

All 106 patients (male: 68, female: 38; mean age, 62.42 ± 10.22 years) were randomly divided into the training cohort (*n* = 74) and the validation cohort (*n* = 32) (the ratio of the training to validation cohort was 7:3). The positive rates of EMVI were 28.4% (21/74) and 28.1% (9/32) in the training and validation cohorts, respectively (Supplementary Figure A2).

There were no significant differences (*P* > 0.05) in the clinical-pathologic characteristics between patients in the training and validation cohorts (Table 2). As shown in Table 3, except for pathological T stage and histological grade, there were no statistically significant differences in other variables between the EMVI-positive and -negative groups (*P* = 0.13–0.838), the EMVI-positive group was significantly more frequent in higher pathological T stage (T3-4) and was associated with increased histological grade when compared with EMVI-negative group.

**Table 2:** Clinical and histologic characteristics of patients in the training and validation cohorts.

**Characteristic**	**Training cohort** **(*****n*** **=** **74)**		**Validation cohort** **(*****n*** **=** **32)**		***P-*value**
	**EMVI(+)** **(*n* = 21)**	**EMVI(–)** **(*n* = 53)**	**EMVI(+)** **(*n* = 9)**	**EMVI(–)** **(*n* = 23)**	
Age, years					0.608
≥60	12 (16.2%)	35 (47.3%)	6 (18.7%)	16 (50%)	
<60	9 (12.2%)	18 (24.3%)	3 (9.4%)	7 (21.9%)	
Sex					0.818
Male	14 (18.9%)	34 (45.9%)	4 (12.5%)	16 (50%)	
Female	7 (9.5%)	19 (25.7%)	5 (15.6%)	7 (21.9%)	
Tumor location					0.169
Upper>10 cm	8 (10.8%)	10 (13.5%)	6 (18.7%)	5 (15.6%)	
Middle 5–10 cm	1 (1.4%)	19 (25.7%)	1 (3.1%)	9 (28.2%)	
Lower<5 cm	12 (16.2%)	24 (32.4%)	2 (6.2%)	9 (28.2%)	
Tumor size (cm), median (range)	3.9 (2.4–11)	4.6 (2.7–9.5)	5.1 (2.8–7)	5.6 (2.9–10)	0.141
Histological grade					0.219
Well differentiated	1 (1.4%)	8 (10.8%)	1 (3.1%)	2 (6.3%)	
Moderately differentiated	12 (16.2%)	38 (51.3%)	7 (21.9%)	17 (53.1%)	
Poorly differentiated	8 (10.8%)	7 (9.5%)	1 (3.1%)	4 (12.5%)	
Histologic tumor stage					0.979
pT0-pT2	1 (1.4%)	33 (44.6%)	1 (3.1%)	8 (25.0%)	
pT3-pT4	20 (27.0%)	20 (27.0%)	8 (25.0%)	15 (46.9%)	
Pathologic lymph node					0.962
N0	9 (12.2%)	33 (44.6%)	2 (6.2%)	16 (50%)	
N1-2	12 (16.2%)	20 (27%)	7 (21.9%)	7 (21.9%)	
CEA level					0.405
<5 ng/mL	12 (16.2%)	36 (48.6%)	3 (9.4%)	15 (46.9%)	
≥5 ng/mL	9 (12.2%)	17 (23%)	6 (18.7%)	8 (25%)	
Ki-67					0.974
<40%	1 (1.4%)	5 (6.8%)	0 (0)	5 (15.6%)	
≥40%	18 (24.3%)	38 (51.3%)	6 (18.7%)	12 (40.6%)	
N/A	2 (2.7%)	10 (13.5%)	3 (9.4%)	6 (18.7%)	

*EMVI, extramural venous invasion; CEA, carcinoembryonic antigen*.

**Table 3:** Univariate and multivariate logistic regression analyses between EMVI-positive and –negative groups in the training cohort.

**Variable**	**Univariate analysis**		***P*-value**	**Multivariate analysis**		***P*-value**
	**β**	**Odds ratio (95% CI)**		**β**	**Odds ratio (95% CI)**	
Age, year (mean ± SD)	−0.377	0.69 (0.24, 1.93)	0.475			
Sex	0.1112	1.12 (0.38, 3.25)	0.838			
Tumor location	0.109	1.12 (0.6, 2.06)	0.727			
Tumor size (cm)	0.1197	1.13 (0.87, 1.46)	0.362			
Histological grade	−0.196	0.3 (0.11, 0.82)	0.019^*^	−0.9723	0.38 (0.13, 1.12)	0.08^‘^
Histologic tumor stage	2.495	12.12 (1.51, 97.37)	0.019^*^	2.2455	9.44 (1.15, 77.8)	0.0369^*^
Pathologic lymph node	0.7885	2.2 (0.79, 6.15)	0.13			
CEA level	0.463	1.59 (0.56, 4.49)	0.383			
Ki-67	−0.1987	0.82 (0.29, 2.34)	0.71			

*Significant variables with P < 0.05 in the univariate analysis were included in the multivariate logistic regression analysis. CI, confidence interval; β is the regression coefficient*.

* *P < 0.05*,

‘ *0.05 < P–value < 0.1*.

### Comparison of DCE-MRI Parameters Between the EMVI-Positive and -Negative Groups

The inter-observer agreement for the quantitative parameters of DCE-MRI measured was good; the ICCs of *K*^*trans*^, Kep, Ve, Vp, iAUC, TTP, max slope, and max concentration were 0.80, 0.85, 0.88, 0.87, 0.90, 0.83, 0.77, and 0.80, respectively. A comparison of the DCE-MRI parameters between the EMVI-positive and -negative groups is shown in Table 4. The *K*^*trans*^ and Ve values in the EMVI-positive group were significantly higher than those in the EMVI-negative group (both *P* = 0.02). There was no significant difference in Kep, Vp, or semiquantitative parameters between the EMVI-positive and -negative groups (*P* = 0.128–0.99).

**Table 4:** Comparison of DCE-MRI parameters between the EMVI-positive and EMVI-negative groups(*x* ± *s*)in the training cohort.

	**EMVI+ (*n* = 21)**	**EMVI– (*n* = 53)**	***P*-value**
K^trans^/min^−1^	1.08 ± 0.946	0.542 ± 0.636	0.02^*^
Kep/min^−1^	1.23 ± 0.728	1.31 ± 0.986	0.99
Ve	0.63 ± 0.304	0.455 ± 0.297	0.02^*^
Vp	0.419 ± 0.365	0.288 ± 0.328	0.128
iAUC	7.27 ± 4.37	6.43 ± 3.08	0.525
TTP	3.17 ± 0.553	3.01 ± 0.786	0.328
Max slope	3.28 ± 1.72	3.4 ± 1.57	0.737
Max concentration	1.86 ± 1.05	1.79 ± 0.792	0.933

*K^trans^, volume transfer constant between the blood plasma and the extracellular extravascular space; Ve, extracellular extravascular space volume fraction; Vp, plasma volume fraction; Kep, rate constant of contrast agent escape from the extracellular extravascular space into the plasma compartment; iAUC, initial area under the enhancement curve; TTP, time to peak*.

* *P < 0.05*.

### Feature Selection and Construction of the Radiomics Signature

**Figure 3:**
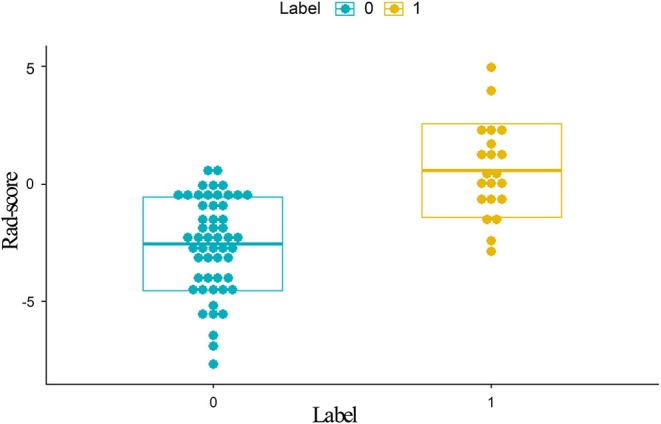
Distribution of scores calculated with the radiomics model from DCE. Label 0 (green dots) represents EMVI-negative patients; Label 1 (yellow dots) represents EMVI-positive patients. EMVI-positive patients generally exhibited higher Rad-scores than EMVI-negative patients. DCE, dynamic contrast-enhanced; EMVI, extramural venous invasion; Rad-scores, radiomics scores.

A total of 792 radiomics features were extracted from T_2_W CUBE and DCE images. Then, these features were reduced to four potential predictors with nonzero coefficients using spearman correlation and LASSO logistic regression analyses, including1 intensity histogram feature, 1 morphological feature, 1 GLCM feature and 1 RLM feature. The Rad-score was calculated for each patient (Figure 3).

Rad-score = −1.73451-0.67785 ^*^ MaxIntensity + 1.81795 ^*^ ClusterProminence_AllDirection_offset7_SD + 1.39861 ^*^ HaralickCOrrelation_AllDirection_offset4 + 1.81083 ^*^ LongRunHighGreyLevelEmphasis_angle0_offset4. The representation of the equation and the meaning of each parameter was presented in the Supplementary Materials.

### Comparison of the Performance of Different Parameters and Prediction Models

The comparisons of predictive performance were evaluated using the DeLong test. There were significant differences amongthe clinical model, quantitative model and R_2_ model in the training and validation cohorts (*P* = 0.0234, clinical model vs. R_2_ mode and *P* = 0.03, quantitative model vs. R_2_ model, respectively). Although there were no significant differences between the R_1_ and R_2_ models (*P* = 0.4807), the predictive performance of the R_2_ model was better than that of the R_1_ model; therefore, the combined model based on DCE with clinical-pathological factors (R_2_-C model) was constructed, and the predictive performance of the R_2_-C model was better than those of the radiomics signature in the validation cohort. There were no significant differences between the R_2_ and R_2_-C models (*P* = 0.3992).

**Figure 4:**
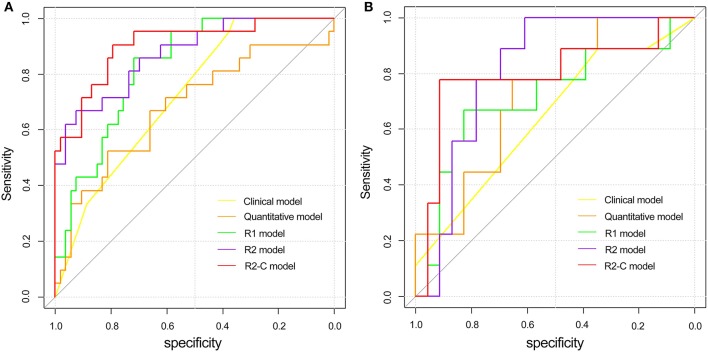
Comparison of ROC curves between the clinical model, quantitative model, and three radiomics models in the training **(A)** and validation **(B)** cohorts. ROC, receiver operating characteristic; R_1_ model indicates the radiomics model based on T_2_W CUBE; R_2_ model indicates the radiomics model based on DCE; R_2_-C model indicates the combined model based on DCE and clinical-pathological factors.

The receiver operating curve (ROC) analysis was used to evaluate the diagnostic performance of each parameter or model for EMVI. *K*^*trans*^ combined with Ve showed a fair degree of accuracy (AUC: 0.680 95% CI, 0.561–0.784) compared with *K*^*trans*^ (AUC: 0.678 95% CI, 0.56–0.782) or Ve (0.670 95% CI, 0.55–0.775) alone, which was validated in the validation cohort (Table 5). Then, *K*^*trans*^ and Ve values were combined to build the quantitative model. As shown in Table 5, the radiomics models (R_1_ and R_2_ models) demonstrated a better diagnostic performance than the clinical and quantitative models. The R_2_-C model exhibited a favorable performance (AUC: 0.904; 95% CI: 0.813–0.96 in the training cohort and AUC: 0.812; 95% CI: 0.635–0.927 in the validation cohort, with a sensitivity of 90.5 and 88.9%, and a specificity of 79.2 and 78.3%, respectively, in both cohorts) and was deemed the optimal model among all the models. ROC curves for the diagnostic performances of the different prediction models were summarized in Figure 4.

**Table 5:** Diagnostic performance of the different parameters and models for predicting EMVI.

**Methods**	**Training cohort (*****n*** **=** **74)**			**Validation cohort (*****n*** **=** **32)**		
	**AUC (95% CI)**	**SEN**	**SPE**	**AUC (95% CI)**	**SEN**	**SPE**
Histological grade	0.65 (0.531–0.758)	0.381	0.868	0.539 (0.354–0.716)	0.889	0.174
T stage	0.665 (0.546–0.77)	0.953	0.377	0.618 (0.43–0.784)	0.889	0.348
Clinical model	0.723 (0.607–0.821)	1.00	0.358	0.643 (0.454–0.803)	0.889	0.348
K^trans^	0.678 (0.56–0.782)	0.524	0.830	0.628 (0.44–0.791)	0.556	0.739
Ve	0.670 (0.55–0.775)	0.809	0.566	0.715 (0.529–0.86)	0.778	0.696
Quantitative model	0.680 (0.561–0.784)	0.524	0.811	0.715 (0.529–0.86)	0.778	0.652
R_1_ model	0.826 (0.720–0.904)	0.857	0.717	0.715 (0.529–0.860)	0.667	0.826
R_2_ model	0.872 (0.774–0.939)	0.667	0.925	0.812 (0.662–0.960)	1.000	0.609
R_2_-C model	0.904 (0.813–0.96)	0.905	0.792	0.812 (0.635–0.927)	0.889	0.783

*The R_1_ model indicates the radiomics model based on T_2_W CUBE; the R_2_ model indicates the radiomics model based on DCE; the R_2_-C model indicates the combined model based on DCE and clinical-pathological factors; the quantitative model indicates the model that combines the K^trans^ and Ve values; SEN, sensitivity; SPE, specificity; AUC, area under the curve; CI, confidence interval*.

### Construction of the Radiomics Nomogram

**Figure 5:**
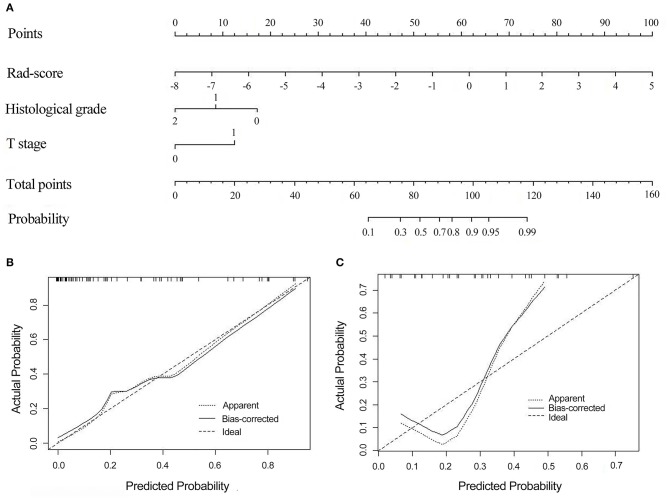
The radiomics nomogram combines three items: the Rad-score, T stage, and histological grade **(A)**. Calibration curves of the radiomics nomogram-based prediction in the training **(B)** and validation **(C)** cohorts. The calibration curves represent the calibration of the nomogram based on agreement between the predicted risk of EMVI and actual EMVI findings. A close fit between the dotted and solid lines indicates good predictive accuracy of the nomogram. Rad-score, radiomics score; EMVI, extramural venous invasion.

The radiomics nomogram was constructed by integrating the optimal radiomics model from DCE with clinical-pathologic features, including histopathological grade and T stage, in the training cohort. The calibration curve for the radiomics nomogram was tested by the Hosmer–Lemeshow test and showed no significant difference (*P* = 0.497 and *P* = 0.107 in the training and validation cohorts, respectively) between the calibration curves and a perfect fit for predicting EMVI (Figure 5).

### Clinical Use

**Figure 6:**
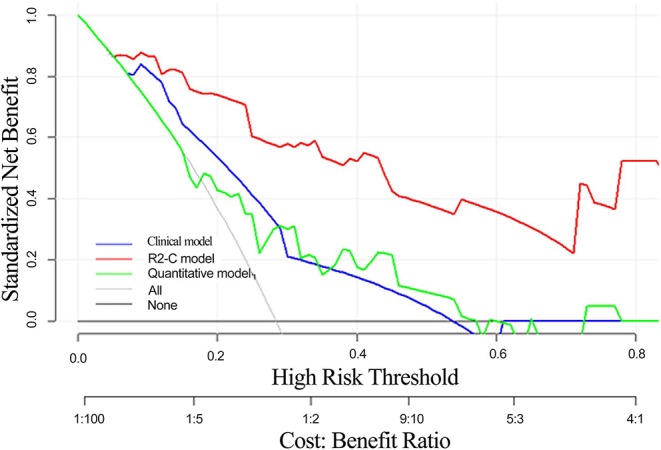
Decision curve analysis of the radiomics nomogram, clinical model, and quantitative model. The y-axis indicates the net benefit. The red line represents the radiomics nomogram. The blue line represents the clinical model. The green line represents the quantitative model. The gray line represents the assumption that all patients have EMVI, and the thin black line represents the assumption that no patient has EMVI.

The decision curve analysis for the radiomics nomogram, the clinical model, and the quantitative model is presented in Figure 6. Decision curve analysis indicates that if the threshold probability of a patient is >10%, using the radiomics nomogram in the current study to predict EMVI adds more benefit than the treat-all-patients scheme or the treat-none scheme. In addition, the clinical-radiomics combined model had the largest overall net benefit compared with the clinical model and the quantitative model across the full range of reasonable threshold probabilities.

## Discussion

EMVI can be used to predict a poor prognosis in rectal cancer, and its identification should be necessary either during preoperative staging or after neoadjuvant treatment. The addition of EMVI assessment to rectal cancer risk stratification will contribute to individual decision-making for offering adjuvant treatment. In this study, we developed radiomics models for predicting EMVI in patients with rectal cancer preoperatively and compared the predictive performance of these models with that of DCE-MRI perfusion parameters. The results showed that the R_2_-C model exhibited better diagnostic accuracy than the DCE-MRI quantitative parameters. Furthermore, the clinical-radiomics nomogram was developed as an individualized and visual tool to provide the estimated probability of EMVI for rectal cancer patients.

According to previous studies, risk factors for EMVI include a large tumor size, a high T stage and lymph node stage. In this study, we confirmed that T stage and histological grade were independent predictors of EMVI, which was partly in agreement with the findings of previous studies (23, 32). However, the tumor size and N stage did not correlate with EMVI in our study. Regarding the N stage, this finding could be explained by selection bias, and the tumor was drawn manually, so it may be affected by individual factors. Therefore, these factors alone should not be used for preoperative decision-making. Chen et al. (33) indicated that the gross tumor volume on DWI and T_2_W images could be used for the preoperative evaluation of lymphovascular invasion (LVI) in rectal cancer and showed favorable AUCs (0.899 and 0.877, respectively), but the volumetric measurement and assessment would be time consuming and may not reflect the intratumoral nature of EMVI; the results also lacked validation. Ahn et al. (34) attempted to assess the potential quantitative method of DWI for evaluating EMVI in rectal cancer; however, the diagnostic performance was not significantly improved when DWI was added for the detection of EMVI.

In the present study, we evaluated the association between DCE-MRI parameters and EMVI in rectal cancer, and only *K*^*trans*^ and Ve showed significant differences between the EMVI-positive and -negative groups. The results showed that the EMVI-positive group with rectal cancer had significantly higher *K*^*trans*^ and Ve values than the EMVI-negative group, and these values were positively correlated with EMVI, suggesting that these two parameters may be closely related to vascular invasion of tumors. Ve is the fractional volume of the EES. During tumor progression, tumor cells secrete vascular endothelial factors that increase the permeability of tumor blood vessels and loss of function of cell-cell adhesion molecules, leading to a large interstitial space and resulting in an enlarged EES (35, 36); thus, it was not surprising that the Ve value was significantly higher in the EMVI-positive group than in the EMVI-negative group. This result was in agreement with those of Chen et al. (23), who reported that MRI-detected EMVI-positive patients exhibited an increased Ve value. Regarding the *K*^*trans*^ value, our finding was consistent with a recent study that showed that LVI was correlated with a high *K*^*trans*^ value in patients with rectal cancer (35) but contrary to that of Chen et al. (23). This may be explained by the different inclusion criteria between our studies. In Chen et al.'s study, the investigation was based on MRI-detected EMVI (rather than EMVI detected on histopathology, as in our study), which may not fully reflect the actual status of EMVI, especially the early stage of rectal carcinoma. In addition, the *K*^*trans*^ value is affected by blood perfusion, including cardiac output, hypertension, and the circulatory system of an individual, which can produce high individual variation. However, in our study, the performances of these two parameters were mediocre, and it is worth noting that when *K*^*trans*^ was combined with Ve, the diagnostic performance of EMVI was not significantly improved compared with that of individual parameters. It is speculated that *K*^*trans*^ and Ve do not increase unlimitedly with the increase in the malignant degree and invasion depth of tumors; when the concentration of contrast agent in the EES reaches a certain degree, the pressure difference inside and outside microvessels decreases, affecting the diffusion rate of contrast agent from microvessels to the EES, and the two may be correlated and restricted.

We further investigated the radiomics features for the prediction of EMVI. Recent studies have reported that the radiomics signature could be used to preoperatively predict microvascular invasion (MVI) in hepatocellular carcinoma and LVI in breast cancer (37, 38). Similarly, our study demonstrated that the radiomics signature was significantly associated with the EMVI status in rectal cancer. The radiomics features, MaxIntensity belongs to histogram features, which indicate the distribution of voxel intensities within the images, ClusterProminence AllDirection offset7_SD belongs to texture feature, reflects the asymmetry of a given distribution, HaralickCorrelation AllDirecion offset4 belongs to GLCM feature, represents the local gray level correlation (the greater its value, the greater the correlation), and the LongRunHighGreyLeveEmphasis angle0_offset4 belongs to the RLM features, which takes into account the length of consecutive pixels or voxels with the same gray values in a specific direction. All the 4 radiomics features were included in our prediction model to build the radiomics signature, which may reflect the different growing patterns and the tumor heterogeneity of rectal cancer. To the best of our knowledge, this is the first time that radiomics was used to evaluate EMVI in rectal cancer, and a nomogram that combines the Rad-score with clinical-pathological factors was constructed to further improve its predictive accuracy for EMVI.

In our study, we constructed radiomics models based on T_2_W CUBE and DCE imaging and a clinical model based on clinical-pathologic risk factors. Our results demonstrated that the R_2_ model performed better than the R_1_ model in both cohorts. On the one hand, because EMVI is associated with angiogenesis, we used the post-contrast images at 60 s, which provide improved tissue contrast for tumor segmentation, considering that these images probably contain more information on intratumoral heterogeneity and allow us to acquire more features concerning the blood supply than unenhanced images for predicting EMVI. On the other hand, it is difficult to differentiate the fibrotic response from the perirectal tumor on T_2_W imaging, whereas DCE-MRI can clearly distinguish the high signal intensity of the tumor or blood vessels from the low signal intensity of the fibrotic response. In addition, the R_2_-C model further improved its predictive performance for EMVI and exhibited an excellent performance to identify high risks of EMVI. The predictive accuracy of the clinical-radiomics model was significantly improved compared with the clinical model alone, indicating that the combined model may have greater value in EMVI prediction than clinical-pathological features. Jiang et al. (39) who also integrated CT-based radiomics features in conjunction with clinical-pathological risk factors, demonstrated a better performance than the use of clinical risk factors alone in predicting lymph node metastasis in gastric cancer. These findings support the idea that the use of different aspects of factors is the most prospective way to assist clinical management (40).

In addition, the combined prediction model developed in our study was presented as an easy-to-use nomogram, which can easily calculate the risk of EMVI tailored to each individual patient and assist in clinical decision-making. This proposed radiomics nomogram may assist in risk stratification for patients and optimize treatment decisions before an operation. Furthermore, even though rectal cancer can be resected to discover early recurrence and metastases, high-risk patients should be carefully monitored postoperatively, and appropriate adjuvant or neoadjuvant therapy is also required to prevent recurrence after resection in these patients.

As mentioned above, all of the radiomics models demonstrated an outperformed predictive performance compared with the quantitative model, which extracted multidimensional imaging features of the whole tumor, thus better reflecting the biological behavior and intratumoral heterogeneity, providing a more accurate, objective, and convenient way to predict EMVI.

There were several limitations to this study worth mentioning. First, we extracted the DCE-MRI parameters from the entire volume of the rectal tumor and obtained the average value of the parameters of the whole tumor. Therefore, the diagnostic efficacy of the parameters may be lower than the actual diagnostic ability of the parameters of the EMVI area. Second, the manual segmentation of ROIs is time consuming and requires the development of automated segmentation techniques when very large databases are evaluated. Third, the sample size of our study was really small (the exclusion of lesions that were too small to be accurately measured, lesions that scored 4 based on the five-point grading system, and the M stage was not considered). Moreover, we selected only one contrast-enhanced phase for tumor image segmentation and feature extraction, which may not completely demonstrate tumor angiogenesis. Additionally, our study lacks external validation for the model and requires further multicentre validation. Finally, radiogenomics, as an emerging field of cancer research, has attracted increasing interest, and it will be interesting for us to explore whether the construction of a radiogenomics model can exhibit a better predictive performance than the radiomics model in the future.

In conclusion, our study demonstrated that the *K*^*trans*^ and Ve values of DCE-MRI quantitative parameters were significantly associated with EMVI, enabling DCE-MRI parameters to serve as an additional tool for potentially predicting EMVI. However, their moderate diagnostic performances may limit their clinical application. The predictive performance of the R_2_-C model was relatively higher than that of the quantitative parameters and superior to that of all the other models. Even with a limited training data set, the diagnostic accuracy of the R_2_-C model seems to reach clinically acceptable levels, especially for suspicious EMVI which scored 2–3 according to the five-score EMVI grading system (31). Furthermore, the non-invasive radiomics nomogram, which includes the Rad-score and clinical-pathologic risk factors, exhibits good application potential in clinical practice in terms of the preoperative individualized prediction of EMVI.

## Data Availability Statement

The datasets generated for this study are available on request to the corresponding author.

## Ethics Statement

This retrospective study was approved by the Medical Ethics Committee of the Second Affiliated Hospital of Chongqing Medical University, and all patients provided written consent for the DCE- MRI examination.

## Author Contributions

XY, DG, XL, and HZ conceived and designed the study. XY, WS, and JS organized the database. HL performed the statistical analysis. XY wrote the first draft of the manuscript. XH and JZ wrote sections of the manuscript. All authors contributed to manuscript revision and read and approved the submitted version.

### Conflict of Interest

HL was employed by the company GE Healthcare, Shanghai, China. The remaining authors declare that the research was conducted in the absence of any commercial or financial relationships that could be construed as a potential conflict of interest.
