# Supplementary material for: Preoperative Prediction of Extramural Venous Invasion in Rectal Cancer: Comparison of the Diagnostic Efficacy of Radiomics Models and Quantitative Dynamic Contrast-Enhanced Magnetic Resonance Imaging
Source: Front Oncol. 2020 Apr 9;10:459. doi: 10.3389/fonc.2020.00459 (PMC7160694; doi:10.3389/fonc.2020.00459)
Supplement: Supplementary file 1 [file Data_Sheet_1.DOCX]

Supplementary Material

**Preoperative prediction of extramural venous invasion in rectal cancer: comparison of the diagnostic efficacy of radiomics models and quantitative dynamic contrast-enhanced magnetic resonance imaging**

Xiangling Yu, BS^1^, Wenlong Song, BS^1^, Dajing Guo, MD^1^, Huan Liu, MD^2^, Haiping Zhang, MS^1^, Xiaojing He, MD^1^, Junjie Song, BS^1^, Jun Zhou, MS^1^, Xinjie Liu, MD^1*^.

^1^ Department of Radiology, the Second Affiliated Hospital of Chongqing Medical University, Chongqing, China

^2^ GE Healthcare, Shanghai, China

***Correspondent author:**

Xinjie Liu, Department of Radiology, the Second Affiliated Hospital of Chongqing Medical University, No. 74 Linjiang Rd., Yuzhong District, 400010 Chongqing, China.

Tel.: +86 23 63693238

1. mail: [302163@hospital.cqmu.edu.cn](mailto:302163@hospital.cqmu.edu.cn)

**[Supplementary](https://www.frontiersin.org/articles/10.3389/fonc.2019.00340/full#SM1) [Figure](https://www.frontiersin.org/articles/10.3389/fonc.2019.01203/full#F1)s:**


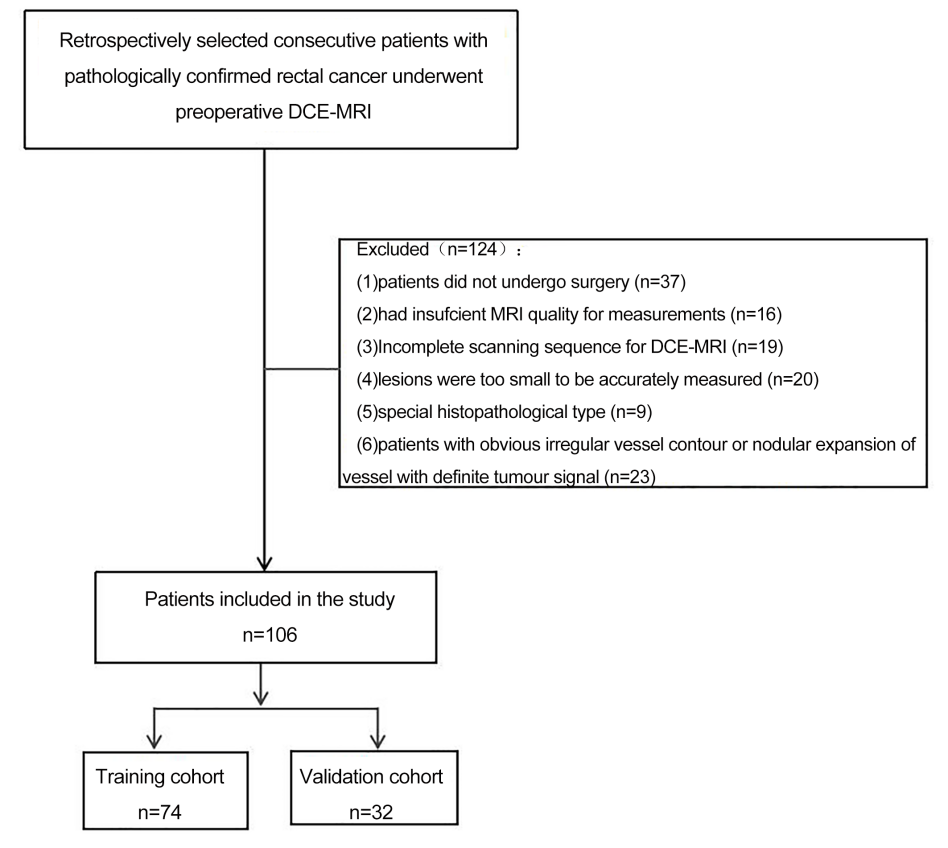


[Supplementary](https://www.frontiersin.org/articles/10.3389/fonc.2019.00340/full#SM1) [Figure](https://www.frontiersin.org/articles/10.3389/fonc.2019.01203/full#F1) A1: Flow diagram of the study patients


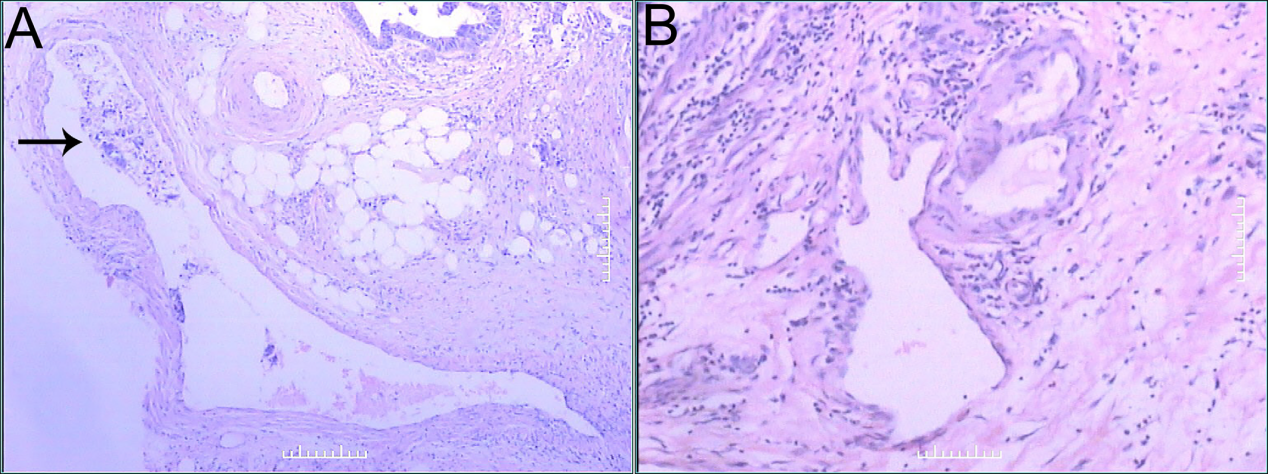


[Supplementary](https://www.frontiersin.org/articles/10.3389/fonc.2019.00340/full#SM1) [Figure](https://www.frontiersin.org/articles/10.3389/fonc.2019.01203/full#F1) A2：Histology of extramural vascular invasion in rectal cancer. A: positive EMVI was confirmed on histopathology. B: negative EMVI was confirmed on histopathology

**[Supplementary](https://www.frontiersin.org/articles/10.3389/fonc.2019.00340/full#SM1) results:**

Rad-score=-1.73451-

0.67785*MaxIntensity+1.81795*ClusterProminence_AllDirection_offset7_SD+1.39861*HaralickCOrrelation_AllDirection_offset4+1.81083*LongRunHighGreyLevelEmphasis_angle0_offset4.

MaxIntensity: Histogram parameters are concerned with properties of individual pixels. They describe the distribution of voxel intensities within the CT image through commonly used and basic metrics. Let *X* denote the three-dimensional image matrix with voxels and the first-order histogram divided by discrete intensity levels. MaxIntensity: the maximum intensity value of *X.*

Cluster Prominence: Cluster Prominence is a measure of asymmetry of a given distribution; high values of this feature indicate that the symmetry of the image is low. in medical imaging, low values of cluster prominence represent a small peak for the image grey-level value, and usually, the grey-level difference between the forms is small. Formula:

$$\sum_{i,j} ((i-\mu)+(j-\mu))^{4}g(i,j)$$

Haralick Correlation: Measures the degree of similarity of the grey level of the image in the row or column direction. Represents the local grey-level correlation; the greater its value is, the greater the correlation. Formula:

$$-\sum_{i,j} \frac{(i,j)g(i,j)-\mu_{t}^{2}}{\sigma_{t}^{2}}$$

* where $\mu_{t}$and $\sigma_{t}$are the mean and standard deviation of the row (or column, due to symmetry) sums.

Long Run High Grey Level Emphasis:

$$LRHGE(\theta)=\frac{1}{n_{r}}\sum_{j=i}^{N} \sum_{j=1}^{M} p(i,j,\theta)i^{2}j^{2}$$

where $n_{r}$is the total number of runs and $n_{p}$is the number of pixels in the image.

And the coefficients were obtained by logistic regression analysis.

**
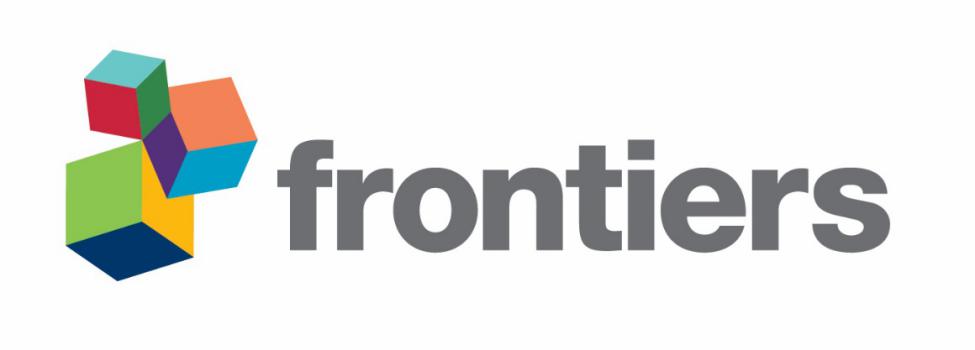
**
